# Neurophysiological mechanisms of embodied empathy in martial arts

**DOI:** 10.3389/fpsyg.2026.1782703

**Published:** 2026-03-12

**Authors:** Guy Shpak

**Affiliations:** Mind-Set Framework, Berkel en Rodenrijs, Netherlands

**Keywords:** embodied empathy, interpersonal synchronisation, martial arts, mirror neuron system, oxytocin, physical contact

## Abstract

Martial arts are increasingly recognized as practices that extend far beyond the development of martial-related physical skills and are commonly used for recreational and even therapeutic purposes. A central construct that is developed in all forms of martial arts is empathy, which shapes the nature of this social interaction. This paper analyzes the intersection between martial arts and empathy and focuses on two interconnected mechanisms associated with both: physical contact and interpersonal synchronization. Physical contact is inherent to all martial arts practices, including grappling, sparring, and partner drills, which engage affective somatosensory touch pathways that are related to empathy. In parallel, interpersonal synchronization stemming from coordinated movement and leading-following dynamics enhances sensorimotor alignment, which is also associated with empathy. By analyzing the shared neurophysiological mechanisms between martial arts practice methodologies and empathy, this paper proposes a model that integrates related neural networks and molecular players that together define a unique neurophysiological “martial arts empathy signature.” Furthermore, by suggesting that empathy can be developed as a transferable skill, and given the regulated and safe social settings martial arts offer, this perspective paper suggests that martial arts practice is an accessible tool for promoting empathy and improving our lives as individuals and societies.

## 1 Introduction

Despite the lack of broad agreement on a comprehensive definition, empathy is acknowledged as being a complex construct central to our social lives. Generally defined, empathy is the ability to understand and potentially identify with others’ cognitive, affective, and sensory states. This ability to “read the mind” of others can be limited to the individual’s internal experiences or result in an interaction with others. Empathy promotes social interactions and prosocial behaviors, which are deeply related to compassion (Elam et al., 2025; Mello et al., 2024; Morelli et al., 2015), and the importance and implications of empathy are relevant in education (Decety, 2020; Marshall and Hooker, 2016; Zhou et al., 2021), medicine (Decety, 2020; Marshall and Hooker, 2016; Zhou et al., 2021), in the context of leadership (Muss et al., 2025; Nakamura et al., 2022), and essentially any other aspect of our social lives. Decades of research depict empathy as a multidimensional construct that involves cognitive and affective processes with distinct behavioral and neural substrates (Cuff et al., 2016; Guthridge and Giummarra, 2021; Heyes, 2018; Smith, 2017). Importantly, research suggests that empathy is fundamentally rooted in bodily dynamics and sensorimotor coupling (Ferrari and Coudé, 2018; Marshall and Hooker, 2016; Moore et al., 2020; Schaefer et al., 2012; Schmidsberger and Löffler-Stastka, 2018). This approach adds to the concept of empathic experience as a multidimensional construct in which somatic processing is deeply intertwined with the cognitive and affective processes. Different lines of research point to several involved systems, including the somatosensory and the mirror neuron systems (Bonini et al., 2022; Ferrari and Coudé, 2018; Jeon and Lee, 2018), as well as underlying physiological mechanisms, which were described in the context of social interactions (Levy et al., 2016; Mu et al., 2016; Procyshyn et al., 2020; Xu et al., 2019). These are instrumental for social interactions that involve physical activity, as in the case of martial arts.

Early records of human fighting systems go back thousands of years, with evidence for practices from all around the globe. Through the years, non-martial aspects percolated these practices, including philosophical, spiritual, and scientific ideas, establishing the transformation from martial systems to martial arts. Modern martial arts practices can be divided into three key domains: self-defense (including military/law enforcement systems, etc.), sports, and recreation. While each domain prioritizes specific desired outcomes, the training approaches are often overlapping, and all share several core pedagogical features (Shpak and Vasques, 2023). Next to acknowledging the physiological benefits of martial arts practice, a growing body of research highlights the unique psychological outcomes of a variety of these practices. Specifically, research found a positive effect of martial arts practice on mood (Jansen et al., 2016; Sharma and Haider, 2015; Zhang et al., 2014), attention (Douris et al., 2015; Johnstone and Marí-Beffa, 2018a), self-esteem (Bao and Jin, 2015), aggressiveness (Lai et al., 2018; Moore et al., 2019), and more, making these arts potential therapeutic interventions (Bu et al., 2010; Ciaccioni et al., 2024; Harwood-Gross et al., 2021a; Martin and Advisor, 2002; Marusak et al., 2022; Moore et al., 2020). Martial arts practice was also found to improve cognitive processes (Fabio and Towey, 2018; Giordano et al., 2021; Harwood-Gross et al., 2021a; Rinderer and Bernero, 2017). Taken together, these can be considered as relevant for both affective and cognitive aspects of empathy.

As increasingly organized methods, martial arts specify codes and morals alongside the physical routines as an integral part of the practice, such as self-discipline, courage, honor, and more. Among these principles, the importance of social responsibility and caring for your fellow human beings is paramount. Importantly, different pedagogical approaches were introduced to develop these social skills, with empathy standing at their core. Given the physical nature of martial arts practice, the associated empathy is highly related to the perception of empathy as an embodied construct. Several components of these practices can be considered in the context of empathy, particularly partner interaction involving direct physical contact and synchronous movement, both are associated with empathy independently of martial arts settings (Ferrari and Coudé, 2018; Goldstein et al., 2017; Jeon and Lee, 2018; Josef et al., 2019; Jospe et al., 2018; Paz et al., 2022; Schmidsberger and Löffler-Stastka, 2018).

## 2 Physical contact and empathy

Touch is a fundamental aspect of human social interaction that is known to activate brain regions and physiological processes that are associated with safety, reward, and social bonding. In non-martial arts settings, physical touch is pivotal in many forms of non-verbal social communication, from expressing support with a casual touch on the shoulder, a handshake, a caring hug, etc. This central role of tactile information on social interaction positions the skin as a social organ (Field, 2010a; Morrison et al., 2010).

All martial arts practices involve typical forms of carefully managed physical interactions, including sparring, grappling, and partner drills that serve predetermined purposes (self-defense scenarios, sportive competition, sensory awareness, etc.). While striking is focused on fast and short bouts of physical contact, grappling and partner drills involve a longer duration of physical contact, which can be considered as an embodied conversation. In Judo, different wrestling and Jiujitsu styles, practitioners are engaged in a prolonged and complex physical contact, in which they are trained to extract a huge amount of information derived from the tactile cues they detect during the bout, with embodied empathy playing a central role (Gravino et al., 2025; Kozdras, 2019). Referring to the cognitive efforts involved, Judo, Jiujitsu in its different forms (from Daito-ryu to Brazilian Jiu-Jitsu, for example), and others, are highly demanding and considered to be a “physical Chess game.” In practices that involve a more defined physical contact, as can be found in partner drills like pushing-hands exercises in Tai Chi, Karate Kaki, and others, the purpose is explicitly directed toward sharpening attentional skills for both the physical and mental states of each other. In all forms of martial arts practices, practitioners use tactile information to be attuned to their partner’s structure, force, timing, rhythm, breath, and other factors, and create an idea, or ‘a feeling’, regarding their intentions, physical and technical abilities and strategies, and others, that may be used in the context of the specific interaction.

Generally, touch is mediated in the periphery by the somatosensory system, which serves three major functions: exteroception and interoception, tuned for the perception of stimuli originating outside and within our bodies, respectively, and proprioception, tuned for the perception of body position and balance. Relevant to martial arts, somatosensory inputs driven by external inputs that relate to empathy are processed by bottom-up sensory pathways and top-down modulation by the central nervous system. The bottom-up exteroception sensory pathways include two major classes of sensory nerve fibres that mediate the peripheral tactile information to the thalamus: (1) myelinated large-diameter A-fibres, and specifically the Aβ subclass fibres innervating mechanoreceptors in the skin, which are responsible for discriminative touch, allowing perception of various properties of objects, and by that, understanding the environment and effectively controlling our movement. (2) Small-diameter unmyelinated C-fibres, which are typically associated with nociception and pain perception. Some C-fibres, called C-tactile (CT) fibres, respond to gentle, slow stroking of the skin, and given the nature of such physical pressure, are an important pathway underlying pleasant social touch. However, deep pressure, which is mediated by Aβ fibres located deep within the skin and other tissues, can also be considered pleasant and calming, especially when experienced in hugs or massage, for example (Case et al., 2020; Mei et al., 2025).

From the thalamus, information is sent to the primary and then secondary somatosensory cortices. The secondary somatosensory cortex plays an important role in integrating basic tactile information into complex representations. However, the perception of the integrated tactile information is modulated by the central nervous system also in a top-down way, which assigns context that is critical to how this touch is interpreted (Handlin et al., 2023). The assignment of context and meaning to sensory inputs is achieved through interactions between the somatosensory cortices and brain regions crucial for processing emotions, including the amygdala, insula, anterior cingulate cortex, and hippocampus, as well as prefrontal and other regions. The emergent functional networks related to the perception and processing of tactile information play a critical role in constructing embodied empathy. Indeed, it has been demonstrated that activation in the somatosensory cortices is linked to empathy personality traits (Gazzola et al., 2006; Schaefer et al., 2020). Gallo et al. showed that somatosensory activation contributes to empathic decision-making by supporting the transformation of observed reactions of affected body parts into perceptions of pain that are necessary for decision-making (Gallo et al., 2018). In line with that, physical touch was found to promote empathy, showing that both actual physical contact and mirroring are relevant for empathy (Field, 2010a; Goldstein et al., 2017; Schaefer et al., 2012).

When considering underlying molecular mechanisms that play a role in both physical contact and empathy, one of the most studied is oxytocin, which is widely implicated in social bonding, trust, and parental and affiliative behaviors. In the periphery, oxytocin is primarily known for its roles in parturition, lactation, and sexual function, although it also plays a role in autonomic functions, such as heart rate, among others. Within the brain, it functions as a neuromodulator involved in social cognition, paternal behaviors, bond formation, emotional regulation, stress and arousal responses, and more. Oxytocin receptors are widely distributed in brain regions associated with emotion and social behavior, including the amygdala, hippocampus, insula, the anterior cingulate cortex, and brainstem autonomic centers (Hasan, 2024; Quintana et al., 2019; Triana-Del Rio et al., 2022), which were mentioned earlier as connected to the secondary somatosensory cortex (Esmaeilou et al., 2022; Newmaster et al., 2020; Zhang et al., 2021). Physical touch was found to induce oxytocin release, driven by somatosensory activation (Field, 2010b; Morris et al., 2021; Uvnäs-Moberg et al., 2014), and martial arts specifically were found to modulate oxytocin release. Rassovsky et al. found that Jiujitsu training significantly increased oxytocin levels immediately after a high-intensity sparring session, with significantly higher oxytocin increase in grappling compared to striking sparring, probably due to the type of physical contact involved (Rassovsky et al., 2019). A follow-up study looked into the differences in hormonal reactivity in low- and high-risk youth and found that low-risk youths had significantly higher salivary oxytocin levels at baseline and at peak-training compared to high-risk youths. Furthermore, low- and high-risk youth showed differences in the reactivity of oxytocin and cortisol secretion (Harwood-Gross et al., 2020). Another study elaborated on these findings and demonstrated correlated cognitive and behavioral changes (Harwood-Gross et al., 2021b).

## 3 Interpersonal synchrony and empathy

By emphasizing timing, rhythm, and shared flow, martial arts provide ideal conditions for interpersonal synchrony at different levels, with at least two aspects of interpersonal synchrony that can be considered: mimicry, and entrainment, or leading-following dynamics. Critically, different forms of interpersonal synchrony were found to be correlated with empathy (Cirelli, 2018; Hove and Risen, 2009). Generally speaking, mimicry in martial arts can be found in the methodologically coordinated practice of basic techniques, as well as in the performance of structured sequences like kata in Japanese arts, taolu in Chinese gong-fu, and others, which rely largely on perception and action matching of a defined movement pattern, when performed as a group. However, these can also be performed as entrained movements, in which one follows a leader’s rhythm. Such leading-following dynamics are considered highly demanding and require more cognitively and sensorimotorically forward modeling, in which one must predict upcoming actions in an unpredictable environment based on incomplete information, a task that requires continuous reciprocal adjustment. Different forms of sparring, grappling, self-defense scenario drills, etc., require fast, online leading-following dynamics analysis of a variety of sensory information, all while keeping in mind a defined desired outcome for this interaction, safety considerations, etc. Whether via mimicry or leading–following dynamics, martial arts practices develop attention to the others’ physical, cognitive, and emotional states, all related to empathy.

Physiological correlates of interpersonal synchrony in mammals can be traced as early as during the mother–infant bond formation (Doi et al., 2011; Feldman, 2007). The coordinated behavior and matched physiological response between parent–infant and later social partners profoundly shape the development of human social connections. The ability to identify with others by motor matching, known as kinesthetic empathy, is one potential link between interpersonal synchrony and empathy. Overall, this ability relies on several neural networks that are referred to as the “social brain” (Adolphs, 2008), including the mirror neuron system, which responds to both action performance and action observation. The mirror neuron system, which was first shown to be involved in movement-related mirroring (Rizzolatti et al., 1996), is also involved in processing emotional expressions of others and empathy, although the evidence for that is mixed (Bekkali et al., 2020; Schmidt et al., 2021). Interestingly, both the amygdala and anterior cingulate cortex, which were mentioned earlier in the context of somatosensation and the oxytocinergic system, were found to be a part of this network.

Oxytocin was also found to play a role in behavioral synchrony, activation of the mirror neuron system, and regulation of autonomic nervous system processes related to social interaction, stress regulation, and more. Research showed that oxytocin facilitates social interaction by enhancing sensorimotor predictions in the context of leader-follower interpersonal synchronisation (Gebauer et al., 2016), and, interestingly, increases movement synchrony in dancing pairs (Josef et al., 2019).

The link between synchrony and empathy is bidirectional, and individuals with higher empathic skills synchronize better with others than those with lower empathy (Bamford and Davidson, 2019; Novembre et al., 2019; Tzanaki et al., 2025). Dancing, which involves both synchronization and exertion in a similar way to martial arts, has a positive effect on social bonding and pain threshold, which is mediated by the endogenous opioid system (Tarr et al., 2015). While endorphin secretion is associated with the feelings of relaxation after hard workouts and the “runner’s high,” for example, it was also found to be involved in synchronized motor behavior. By measuring the change in pain threshold as a proxy for endorphin release, Tarr et al.’s findings suggest that the endogenous opioid system is activated during synchronous activities independently of the level of exertion, making this system another potential link between behavioral synchrony itself and social bonding.

One can argue that, as with other learned skills, the functional connectivity of neural networks that integrate somatosensory, affective, and cognitive processes related to empathy can be trained and strengthened by consistently activating them during synchronized social activities, and especially activities that involve mirroring several sensory modalities, as in martial arts. Indeed, research showed that martial arts practice is associated with improvements in the attentional brain network in an experience-dependent manner (Johnstone and Marí-Beffa, 2018b).

## 4 Discussion

Martial arts provide unique methodologies where neurophysiological mechanisms of physical interactions converge on empathy. When practiced under clear rules and boundaries and in a mindful way, engaging in these physical interactions can be perceived as a continuum between direct physical contact and an extended interpersonal synchrony, with shared underlying neurophysiological mechanisms. It is important to note that this continuum reflects a deep connection between physical contact and interpersonal synchronization, in which the combative aspect of martial arts is maintained in both, even though individual practices like kata/taulu do not involve direct physical contact. In all individual practices, practitioners are encouraged to internally simulate a combative-related aspect, whether as an imagined combat or allocate attention to martial-related bodily states (breathing, skeletal alignment, muscle tension, etc.).

A model that describes the neurophysiological mechanisms of embodied empathy in martial arts starts with multimodal perception of sensory inputs that provide context and stimulation. Following initial processing, the sensory information activates several neural networks, including the externally-focused Central Autonomic Network that generates embodied feeling, the internally-focused Default Mode Network that integrates the information into self-other narrative, and the Salience Network, which evaluates relevance and moderates the two. The Mirror Neuron System operates within the crosstalk of these networks, providing a “somatosensory scaffold” for the other mentioned networks, which is critical not only for the somatosensory, but also the cognitive and affective identification with our social environment. The balance between the martial purposes and social attunement is evident in the combination of molecular players that might seem contradictory, including increased secretion of oxytocin and endorphins, but also cortisol (Harwood-Gross et al., 2020). Together, this combination provides a unique “physiological martial arts signature” that modulates the activity of these networks in a bidirectional manner. Specifically, several forms of empathy emerge from these functional and physiological states, as evident in the shared mechanisms between physical touch, interpersonal synchrony, and empathy.

I argue that by providing combative-oriented stimulation in a safe social context, which includes an embodied affective and cognitive experience on a continuum between direct somatosensory-mediated contact and indirect interpersonal synchronization, martial arts offer a unique type of social interaction that shapes our sense of empathy in distinct ways. Moreover, when practiced persistently, the observed network modulations, from functional connectivity to structural modifications and molecular reactivity, provide a typical neurophysiological state that may facilitate the transferability of embodied empathy to other social contexts, and some preliminary work supports this idea (Katz, 2024; Kozdras, 2019).

Since interactive forms of martial arts practices are strictly regulated, and individual forms are introspective by nature, these practices offer a safe social settings that gain an increased recognition for their potential to promote physiological, cognitive, affective, and social aspects of our lives, and are accordingly perceived as much more than sports activities.

At the heart of these practices is a social form of physical activity with varied and regulated levels of physical and mental arousal, which are closely related to empathy.

By accounting for the embodied nature of empathy and looking into potential underlying mechanisms, we can tune these practices to further develop empathy as a transferable skill. This can be done by attention to the ways physical contact and interpersonal synchrony are trained within martial arts schools, but also by offering ways to integrate these in non-martial arts settings, using martial arts concepts. Exercises variations of pushing hands may offer a safe, consensual way to use physical contact, and simplified group kata/Qigung practice may offer an opportunity to move in sync. Such exercises can be practiced anywhere, without any required equipment or specialized space, and can be modified to fit any target group.

## Data Availability

The original contributions presented in the study are included in the article/supplementary material, further inquiries can be directed to the corresponding author.

## References

[ref1] Adolphs R. (2008). The Social Brain: Neural Basis of Social Knowledge. 10.1146/annurev.psych.60.110707.163514 PMC2588649 18771388

[ref2] Bamford J. M. S. Davidson J. W. (2019). Trait empathy associated with agreeableness and rhythmic entrainment in a spontaneous movement to music task: preliminary exploratory investigations. Musicae Sci. 23, 5–24. doi: 10.1177/1029864917701536

[ref3] Bao X. Jin K. (2015). The beneficial effect of tai chi on self-concept in adolescents. Int. J. Psychol. 50, 101–105. doi: 10.1002/ijop.12066, 25721879

[ref4] Bekkali S. Youssef G. J. Donaldson P. H. Albein-Urios N. Hyde C. Enticott P. G. (2020). Is the putative mirror neuron system associated with empathy? A systematic review and meta-analysis. Neuropsychol. Rev. 31, 14–57. doi: 10.1007/S11065-020-09452-6 32876854

[ref5] Bonini L. Rotunno C. Arcuri E. Gallese V. (2022). Mirror neurons 30 years later: implications and applications. Trends Cogn. Sci. 26, 767–781. doi: 10.1016/J.TICS.2022.06.003, 35803832

[ref6] Bu B. Haijun H. Yong L. Chaohui Z. Xiaoyuan Y. Singh M. F. (2010). Effects of martial arts on health status: a systematic review. J. Evid. Based Med. 3, 205–219. doi: 10.1111/J.1756-5391.2010.01107.X 21349072

[ref7] Case L. K. Liljencrantz J. McCall M. V. Bradson M. Necaise A. Tubbs J. . (2020). Pleasant deep pressure: expanding the social touch hypothesis. Neuroscience 464:3. doi: 10.1016/j.neuroscience.2020.07.050 32768616 PMC7865002

[ref8] Ciaccioni S. Castro O. Bahrami F. Tomporowski P. D. Capranica L. Biddle S. J. H. . (2024). Martial arts, combat sports, and mental health in adults: a systematic review. Psychol. Sport Exerc. 70:102556. doi: 10.1016/J.PSYCHSPORT.2023.102556, 37949383

[ref9] Cirelli L. K. (2018). How interpersonal synchrony facilitates early prosocial behavior. Curr. Opin. Psychol. 20, 35–39. doi: 10.1016/J.COPSYC.2017.08.009, 28830004

[ref10] Cuff B. M. P. Brown S. J. Taylor L. Howat D. J. (2016). Empathy: a review of the concept. Emot. Rev. 8, 144–153. doi: 10.1177/1754073914558466

[ref11] Decety J. (2020). Empathy in medicine: what it is, and how much we really need it. Am. J. Med. 133, 561–566. doi: 10.1016/J.AMJMED.2019.12.012, 31954114

[ref12] Doi H. Kato M. Nishitani S. Shinohara K. (2011). Development of synchrony between activity patterns of mother–infant pair from 4 to 18 months after birth. J. Physiol. Sci. 61, 211–216. doi: 10.1007/S12576-011-0138-Y, 21424393 PMC10717710

[ref13] Douris P. Douris C. Balder N. Lacasse M. Rand A. Tarapore F. . (2015). Martial art training and cognitive performance in middle-aged adults. J. Hum. Kinet. 47, 277–283. doi: 10.1515/hukin-2015-0083, 26672872 PMC4633263

[ref14] Elam T. Efthemiou A. Taku K. (2025). The association positive and negative empathy have with depressive symptoms, resilience, and posttraumatic growth. Sci. Rep. 15:9464. doi: 10.1038/s41598-025-86285-4, 40108175 PMC11923043

[ref15] Esmaeilou Y. Tamaddonfard E. Erfanparast A. Soltanalinejad-Taghiabad F. (2022). Behavioral and receptor expression studies on the primary somatosensory cortex and anterior cingulate cortex oxytocin involvement in modulation of sensory and affective dimensions of neuropathic pain induced by partial sciatic nerve ligation in rats. Physiol. Behav. 251:113818. doi: 10.1016/J.PHYSBEH.2022.113818, 35443199

[ref16] Fabio R. A. Towey G. E. (2018). Cognitive and personality factors in the regular practice of martial arts. J. Sports Med. Phys. Fitness 58, 933–943. doi: 10.23736/S0022-4707.17.07245-0, 28480690

[ref17] Feldman R. (2007). Parent–infant synchrony. Curr. Dir. Psychol. Sci. 16, 340–345. doi: 10.1111/J.1467-8721.2007.00532.X, 17355401

[ref18] Ferrari P. F. Coudé G. (2018). “Mirror neurons, embodied emotions, and empathy,” in Neuronal Correlates of Empathy: From Rodent to Human, (67–77. doi: 10.1016/B978-0-12-805397-3.00006-1

[ref19] Field T. (2010). Touch for socioemotional and physical well-being: a review. Dev. Rev. 30, 367–383. doi: 10.1016/J.DR.2011.01.001

[ref20] Gallo S. Paracampo R. Müller-Pinzler L. Severo M. C. Blömer L. Fernandes-Henriques C. . (2018). The causal role of the somatosensory cortex in prosocial behaviour. eLife 7. doi: 10.7554/ELIFE.32740, 29735015 PMC5973831

[ref21] Gazzola V. Aziz-Zadeh L. Keysers C. (2006). Empathy and the somatotopic auditory mirror system in humans. Curr. Biol. 16, 1824–1829. doi: 10.1016/j.cub.2006.07.072 16979560

[ref22] Gebauer L. Witek M. A. G. Hansen N. C. Thomas J. Konvalinka I. Vuust P. (2016). Oxytocin improves synchronisation in leader-follower interaction. Sci. Rep. 6:38416-. doi: 10.1038/srep38416, 27929100 PMC5144006

[ref23] Giordano G. Gómez-López M. Alesi M. (2021). Sports, executive functions and academic performance: a comparison between martial arts, team sports, and sedentary children. Int. J. Environ. Res. Public Health 18. doi: 10.3390/IJERPH182211745, 34831501 PMC8622860

[ref24] Goldstein P. Weissman-Fogel I. Shamay-Tsoory S. G. (2017). The role of touch in regulating inter-partner physiological coupling during empathy for pain. Sci. Rep. 7, 1–12. doi: 10.1038/s41598-017-03627-7, 28607375 PMC5468314

[ref25] Gravino G. Scala G. Di Palma D. Tafuri F. (2025). Judo as a didactic tool for emotional growth: anger management and positive youth relationships. J. Hum. Sport Exerc. 20, 1402–1412. doi: 10.55860/CPX2HZ67

[ref26] Guthridge M. Giummarra M. J. (2021). The taxonomy of empathy: a meta-definition and the nine dimensions of the empathic system. J. Humanist. Psychol. 65. doi: 10.1177/00221678211018015

[ref27] Handlin L. Novembre G. Lindholm H. Kämpe R. Paul E. Morrison I. (2023). Human endogenous oxytocin and its neural correlates show adaptive responses to social touch based on recent social context. eLife 12. doi: 10.7554/ELIFE.81197, 37157840 PMC10168694

[ref28] Harwood-Gross A. Lambez B. Feldman R. Zagoory-Sharon O. Rassovsky Y. (2021). The effect of martial arts training on cognitive and psychological functions in at-risk youths. Front. Pediatr. 9. doi: 10.3389/FPED.2021.707047, 34746050 PMC8570107

[ref29] Harwood-Gross A. Feldman R. Zagoory-Sharon O. Rassovsky Y. (2020). Hormonal reactivity during martial arts practice among high-risk youths. Psychoneuroendocrinology 121:104806. doi: 10.1016/J.PSYNEUEN.2020.104806, 32721538

[ref30] Hasan R. (2024). The multifaceted role of Oxytocinergic system and OXTR Gene. Global Medical Genetics 11, 29–33. doi: 10.1055/S-0044-1779039, 38239807 PMC10796195

[ref31] Heyes C. (2018). Empathy is not in our genes. Neurosci. Biobehav. Rev. 95, 499–507. doi: 10.1016/j.neubiorev.2018.11.001, 30399356

[ref32] Hove M. J. Risen J. L. (2009). It’s all in the timing: interpersonal synchrony increases affiliation. Soc. Cogn. 27, 949–960. doi: 10.1521/SOCO.2009.27.6.949

[ref33] Jansen P. Dahmen-zimmer K. Kudielka B. M. Schulz A. (2016). Effects of karate training versus mindfulness training on emotional well-being and cognitive performance in later life. Res. Aging 39, 1118–1144. doi: 10.1177/0164027516669987, 27688143

[ref34] Jeon H. Lee S. H. (2018). From neurons to social beings: short review of the mirror neuron system research and its socio-psychological and psychiatric implications. Clin. Psychopharmacol. Neurosci. 16:18. doi: 10.9758/CPN.2018.16.1.18 29397663 PMC5810456

[ref35] Johnstone A. Marí-Beffa P. (2018). The effects of martial arts training on attentional networks in typical adults. Front. Psychol. 9, 1–9. doi: 10.3389/fpsyg.2018.00080, 29472878 PMC5809487

[ref36] Josef L. Goldstein P. Mayseless N. Ayalon L. Shamay-Tsoory S. G. (2019). The oxytocinergic system mediates synchronized interpersonal movement during dance. Sci. Rep. 9, 1–8. doi: 10.1038/s41598-018-37141-1, 30760751 PMC6374432

[ref37] Jospe K. Flöe A. Lavidor M. (2018). The interaction between embodiment and empathy in facial expression recognition. Soc. Cogn. Affect. Neurosci. 13, 203–215. doi: 10.1093/SCAN/NSY005, 29378022 PMC5827354

[ref38] Katz P. (2024). Movements that matter: how martial arts studies can reframe the ethics of first-blush empathy. Martial Arts Studies 2024, 72–83. doi: 10.18573/MAS.185

[ref39] Kozdras G. (2019). Empathy in children practising judo compared to their non-practicing peers. Revista de Artes Marciales Asiáticas 14:40. doi: 10.18002/RAMA.V14I2S.5950

[ref40] Lai A. Fung C. Ka T. Lee H. (2018). Effectiveness of Chinese martial arts and philosophy to reduce reactive and proactive aggression in schoolchildren. J. Dev. Behav. Pediatr. 39, 404–414. doi: 10.1097/DBP.0000000000000565 29649022

[ref41] Levy J. Goldstein A. Zagoory-Sharon O. Weisman O. Schneiderman I. Eidelman-Rothman M. . (2016). Oxytocin selectively modulates brain response to stimuli probing social synchrony. NeuroImage 124, 923–930. doi: 10.1016/J.NEUROIMAGE.2015.09.066, 26455794

[ref42] Marshall G. R. E. Hooker C. (2016). Empathy and affect: what can empathied bodies do? Med. Humanit. 42, 128–134. doi: 10.1136/MEDHUM-2015-010818, 26856355

[ref43] Martin R. A. Advisor I. O.-N. (2002). The physical and psychological benefits of martial arts training for individuals with disabilities. Available online at: http://citeseerx.ist.psu.edu/viewdoc/download?doi=10.1.1.390.2841&rep=rep1&type=pdf

[ref44] Marusak H. A. Borg B. Morales A. Carrington Smith J. Blankenship K. Allen J. L. . (2022). Martial arts-based curriculum reduces stress, emotional, and behavioral problems in elementary schoolchildren during the COVID-19 pandemic: a pilot study. Mind Brain Educ. 16, 5–12. doi: 10.1111/mbe.12307, 35669694 PMC9165706

[ref45] Mei Y. Zhou B. L. Zhong D. Zheng X. J. Deng Y. T. Yu L. . (2025). From sensation to regulation: the diverse functions of peripheral sensory nervous system. Front. Immunol. 16:1575917. doi: 10.3389/fimmu.2025.1575917 40453085 PMC12123694

[ref46] Mello M. Fusaro M. Aglioti S. M. (2024). The neuroscience of human empathy for pleasure: protocol for a scoping review. Syst. Rev. 13:82. doi: 10.1186/S13643-024-02481-9, 38431698 PMC10908019

[ref47] Moore B. Dudley D. Woodcock S. (2020). The effect of martial arts training on mental health outcomes: a systematic review and meta-analysis. J. Bodyw. Mov. Ther. 24, 402–412. doi: 10.1016/j.jbmt.2020.06.017, 33218541

[ref48] Moore B. Woodcock S. Dudley D. (2019). Developing wellbeing through a randomised controlled trial of a martial arts based intervention: an alternative to the anti-bullying approach. Int. J. Environ. Res. Public Health, 16, 1–18. doi: 10.3390/ijerph16010081 PMC6338895 30597946

[ref49] Morelli S. A. Lieberman M. D. Zaki J. (2015). The emerging study of positive empathy. Soc. Personal. Psychol. Compass 9, 57–68. doi: 10.1111/SPC3.12157

[ref50] Morris A. R. Turner A. Gilbertson C. H. Corner G. Mendez A. J. Saxbe D. E. (2021). Physical touch during father-infant interactions is associated with paternal oxytocin levels. Infant Behav. Dev. 64:101613. doi: 10.1016/J.INFBEH.2021.101613, 34311178

[ref51] Morrison I. Löken L. S. Olausson H. (2010). The skin as a social organ. Exp. Brain Res. 204, 305–314. doi: 10.1007/s00221-009-2007-y, 19771420

[ref52] Mu Y. Guo C. Han S. (2016). Oxytocin enhances inter-brain synchrony during social coordination in male adults. Soc. Cogn. Affect. Neurosci. 11, 1882–1893. doi: 10.1093/scan/nsw106, 27510498 PMC5141958

[ref53] Muss C. Tüxen D. Fürstenau B. (2025). Empathy in leadership: a systematic literature review on the effects of empathetic leaders in organizations. Manag. Rev. Q. 76, 1–37. doi: 10.1007/s11301-024-00472-7

[ref54] Nakamura Y. T. Milner J. Milner T. (2022). Inclusive-empathy in leadership. J. Appl. Behav. Sci. 58, 161–163. doi: 10.1177/0021886320982022

[ref55] Newmaster K. T. Nolan Z. T. Chon U. Vanselow D. J. Weit A. R. Tabbaa M. . (2020). Quantitative cellular-resolution map of the oxytocin receptor in postnatally developing mouse brains. Nature Communications 11:1885. doi: 10.1038/s41467-020-15659-1, 32313029 PMC7171089

[ref56] Novembre G. Mitsopoulos Z. Keller P. E. (2019). Empathic perspective taking promotes interpersonal coordination through music. Sci. Rep. 9:12255. doi: 10.1038/s41598-019-48556-9, 31439866 PMC6706439

[ref58] Paz L. V. Viola T. W. Milanesi B. B. Sulzbach J. H. Mestriner R. G. Wieck A. . (2022). Contagious depression: automatic mimicry and the mirror neuron system - a review. Neurosci. Biobehav. Rev. 134:104509. doi: 10.1016/J.NEUBIOREV.2021.12.032, 34968526

[ref59] Procyshyn T. L. Watson N. V. Crespi B. J. (2020). Experimental empathy induction promotes oxytocin increases and testosterone decreases. Horm. Behav. 117:104607. doi: 10.1016/J.YHBEH.2019.104607, 31654674

[ref60] Quintana D. S. Rokicki J. van der Meer D. Alnæs D. Kaufmann T. Córdova-Palomera A. . (2019). Oxytocin pathway gene networks in the human brain. Nat. Commun. 10:668. doi: 10.1038/s41467-019-08503-8, 30737392 PMC6368605

[ref61] Rassovsky Y. Harwood A. Zagoory-Sharon O. Feldman R. (2019). Martial arts increase oxytocin production. Sci. Rep. 2019, 1–8. doi: 10.1038/s41598-019-49620-0, 31506582 PMC6736948

[ref62] Rinderer M. Bernero A. (2017). Mental skills training in martial arts. Available online at: https://digitalcommons.du.edu/capstone_masters/242

[ref63] Rizzolatti G. Fadiga L. Gallese V. Fogassi L. (1996). Premotor cortex and the recognition of motor actions. Cogn. Brain Res. 3, 131–141. doi: 10.1016/0926-6410(95)00038-0, 8713554

[ref64] Schaefer M. Cherkasskiy L. Denke C. Spies C. Song H. Malahy S. . (2020). Empathy-related brain activity in somatosensory cortex protects from tactile priming effects: a pilot study. Front. Hum. Neurosci. 14:500455. doi: 10.3389/fnhum.2020.00142 PMC7253710 32528260

[ref65] Schaefer M. Heinze H. J. Rotte M. (2012). Embodied empathy for tactile events: Interindividual differences and vicarious somatosensory responses during touch observation. NeuroImage 60, 952–957. doi: 10.1016/J.NEUROIMAGE.2012.01.112, 22306799

[ref66] Schmidsberger F. Löffler-Stastka H. (2018). Empathy is proprioceptive: the bodily fundament of empathy - a philosophical contribution to medical education. BMC Med. Educ. 18:69. doi: 10.1186/s12909-018-1161-y 29622015 PMC5887217

[ref67] Schmidt S. N. L. Hass J. Kirsch P. Mier D. (2021). The human mirror neuron system—a common neural basis for social cognition? Psychophysiology 58:e13781. doi: 10.1111/PSYP.13781, 33576063

[ref68] Sharma M. Haider T. (2015). Tai chi as an alternative and complimentary therapy for anxiety: a systematic review. J. Evid.-Based Complement. Altern. Med 20, 143–153. doi: 10.1177/2156587214561327 25488322

[ref69] Shpak G. Vasques A. (2023). Being the finger pointing to the moon: how martial arts can provide a holistic perspective on teachers’ role in tackling sustainability challenges. Front. Educ. 8:1170371. doi: 10.3389/FEDUC.2023.1170371

[ref70] Smith J. (2017). What is empathy for? Synthese 194, 709–722. doi: 10.1007/s11229-015-0771-8, 32226174 PMC7089681

[ref71] Tarr B. Launay J. Cohen E. Dunbar R. (2015). Synchrony and exertion during dance independently raise pain threshold and encourage social bonding. Biol. Lett. 11:20150767. doi: 10.1098/RSBL.2015.0767, 26510676 PMC4650190

[ref72] Triana-Del Rio R. Ranade S. Guardado J. LeDoux J. Klann E. Shrestha P. (2022). The modulation of emotional and social behaviors by oxytocin signaling in limbic network. Front. Mol. Neurosci. 15:1002846. doi: 10.3389/fnmol.2022.1002846 36466805 PMC9714608

[ref73] Tzanaki P. Eerola T. Timmers R. (2025). Actions and feelings in sync: exploring the relationship between synchrony and empathy in children’s dyadic musical interactions. Front. Psychol. 16:1467767. doi: 10.3389/fpsyg.2025.1467767 40351587 PMC12063534

[ref74] Uvnäs-Moberg K. Handlin L. Petersson M. (2014). Self-soothing behaviors with particular reference to oxytocin release induced by non-noxious sensory stimulation. Front. Psychol. 5:116675. doi: 10.3389/fpsyg.2014.01529 PMC4290532 25628581

[ref75] Xu L. Becker B. Kendrick K. M. (2019). Oxytocin facilitates social learning by promoting conformity to trusted individuals. Front. Neurosci. 13, 1–10. doi: 10.3389/fnins.2019.00056, 30787864 PMC6372972

[ref76] Zhang J. Li S. J. Miao W. Zhang X. Zheng J. J. Wang C. . (2021). Oxytocin regulates synaptic transmission in the sensory cortices in a developmentally dynamic manner. Front. Cell. Neurosci. 15:673439. doi: 10.3389/fncel.2021.673439 34177467 PMC8221398

[ref77] Zhang X. Ni X. Chen P. (2014). Study about the effects of different fitness sports on cognitive function and emotion of the aged. Cell Biochem. Biophys. 70, 1591–1596. doi: 10.1007/s12013-014-0100-8, 24997050

[ref78] Zhou Y. C. Tan S. R. Tan C. G. H. Ng M. S. P. Lim K. H. Tan L. H. E. . (2021). A systematic scoping review of approaches to teaching and assessing empathy in medicine. BMC Med. Educ. 21:292. doi: 10.1186/S12909-021-02697-6, 34020647 PMC8140468

